# Dietary change, noncommunicable disease and local knowledge: results of a small-scale study of the views of older Malawians

**DOI:** 10.12688/wellcomeopenres.14887.1

**Published:** 2018-12-11

**Authors:** Megan Vaughan, Albert Dube, Hazel Namadingo, Amelia Crampin, Levie Gondwe, Green Kapira, Joyce Mbughi, Maisha Nyasulu

**Affiliations:** 1 Institute of Advanced Studies, University College London, London, WC1E 6BT, UK; 2 Malawi Epidemiology and Intervention Research Unit, MEIRU/KPS, Lilongwe, PO Box 148, Malawi; 3 Department of Infectious Disease Epidemiology, London School of Hygiene & Tropical Medicine, London, WC1E 7HT, UK

**Keywords:** Malawi, nutrition, agriculture, NCDs

## Abstract

Interviews were conducted with a small group of Malawians over the age of 60 in rural Karonga district and in Area 25 of the capital, Lilongwe. We asked their views on the changes in diet that had taken place over their lifetimes and also on the causes of 'noncommunicable' diseases, such as Type 2 diabetes and hypertension in their communities. Their answers generally confirmed research showing that dietary diversity is decreasing in Malawi, but many of our interviewees also recalled that hunger was more frequently experienced in the past. Our interviews revealed that though the essential rural diet based on either maize or cassava appears superficially largely unchanged, there have been significant changes in the varieties of crops grown, methods of production and food processing. Many of our interviewees were concerned that the application of chemical fertiliser and pesticides was harming their health.

## Introduction

The rising incidence of chronic non-communicable diseases (NCDs) in many sub-Saharan African countries has brought a new focus on ‘lifestyle’, including diet, as a factor in the development of Type 2 diabetes, hypertension and other conditions. In many parts of the continent, high rates of urbanisation, combined with the increasing consumption of processed foods with high salt and sugar content, and home consumption of fried foods and added salt and sugar, replicate the conditions seen elsewhere in the world which have created epidemics of type 2 diabetes and high rates of cardiovascular disease. ( Dalal *et al*., 2011; Nyirenda, 2016; NCD Risk Factor Collaboration, 2017). In a context such as that of Malawi, one of the poorest countries on the continent and with lower rates of urbanisation than most, it is important to acknowledge that ‘lifestyle’ change is almost certainly not the only factor at work. Emerging research points to important interactions between chronic NCDs and infectious diseases and the ‘developmental origins of health and disease’ thesis, combined with work in epigenetics, indicates the ways in which undernutrition, repeated episodes of infectious disease, and other insults before birth and in infancy can have a lasting influence on adult health, including the development of type 2 diabetes, and may cross generations. ( Lelijveld *et al*., 2016; Nyirenda, 2016; Vaughan, 2018). Both of these approaches appear highly relevant to the Malawian context, indicating that there may be multiple ‘routes’ to the development of NCDs in adulthood. In addition, research being conducted by the Lancet Commission on NCDIs and Poverty directs our attention to the role played by inequality and poverty in this complex picture ^1^.

Nevertheless, it seems likely that dietary change, even when not apparently dramatic, plays a role in the complex causation pathways of NCDs in contexts such as that of Malawi. In some cases (that of the urban middle class for example), it may be *the* major factor in the rise of obesity and type 2 diabetes. In other groups, including people with largely agrarian livelihoods, it is possible that even a relatively small change in diet may act as a ‘trigger’, interacting with either a ‘developmental’ susceptibility such as malnutrition in infancy, or an infection such as HIV or tuberculosis. Though new, processed foods are widely available in Malawi as elsewhere in the region, and are particularly attractive to younger people, their penetration into the food system, especially in rural areas, is less deep than in other parts of the southern African region ( Kalimbira & Gondwe, 2015; Mphwante *et al*., 2017). However, even diets that appear relatively unchanged from thirty or forty years ago, may have undergone significant alterations – for example through the adoption of new varieties of staple crops such as maize and cassava, and new methods of preparation. In Malawi, as elsewhere, there is growing concern that diets are becoming less diverse, leading to micronutrient deficiencies, and as such a high proportion of the population is engaged in agricultural production for subsistence, it seems imperative that there is an articulation between agricultural policy (sometimes accused of encouraging monocropping) and nutrition policy which stresses the importance of diversity ( Bezner Kerr, 2013; Gronemeyer *et al*., 2015; Haron, 2018; Jones *et al*., 2014; Kankwamba *et al*., 2018; Koppmair *et al*., 2017; Patel *et al*., 2014).

To learn more about dietary change and perceptions of its relationship to health, we conducted a small-scale exploratory study in rural Karonga district in the north of the country, and in a working class area of the capital, Lilongwe. The aim was modest: to gather the views of older people (60 and over) on questions of changing food production, diet, nutrition and eating patterns. Malawi was the site of extensive nutritional research in the 1930s and 1940, so to some extent it would be possible to compare the results of current research with the observations made then and in intervening decades ( Berry & Petty eds. 1992; Brantley, 2002). Though in no way conclusive, the interviews produced interesting insights into how older people perceive changes in diet in their lifetimes (both positive and negative) and point to questions which might usefully form the basis of more intensive qualitative research.

## Methods

We conducted open-ended interviews with eight individuals in rural Karonga (-10.00.0.00 S 33.44.69.99E) district and seven in urban Area 25 of Lilongwe (13.52.43.3 S 33.43.27.3 E). The Study took place between 15/8/17 and 30/9/17. The majority (11) respondents were female. Though we thought it important to interview both men and women, we found that women were in general more forthcoming on questions of food, especially in relation to processing and preparation.

All of the informants were identified from the database held by the Malawi Epidemiology and intervention Research Unit and had participated in earlier surveys. Some had participated in a recent survey on non-communicable disease. They were all over 60 years of age, but otherwise their selection was based largely on availability and willingness to participate. Their ages ranged from 60 to late 80’s. All were given a written and verbal explanation of the study protocol and all gave consent to take part in the study and to have their views recorded. The study received ethical approval from the National Commission for Science and Technology, Malawi (No. P.07/16/115) and from University College London (No. 7875/002). Interviews were conducted by MN, GK, JM and LG. MV was present at five of the interviews. Audio recordings were made of all the interviews in local languages (chiTimbuka and chiChewa) and transcribed and translated them into English. The interviews were guided by a set of questions discussed in advance, but were open-ended, allowing informants to expand on questions that interested them, or introduce new topics. We began by collecting a basic life history – where the person was born, their movements over their lifetime and their family situation. We asked about level of schooling, marital and work histories and current living situation. This was followed by a set of open-ended questions designed to elicit views on how food production, processing and consumption had changed over their lifetimes. This included questions on cropping patterns, methods of food preparation and the social organisation of eating. We also asked whether they or their family members had ever experienced hunger or had been affected by malnutrition and whether these problems were more or less common today or in the past. We asked them to recall foods that were eaten in the past (referring in particular to their memories of childhood) but which are not seen today, and to talk about the introduction of new crops, new processed foods and beverages. The interviews were concluded by questions relating to obesity, type 2 diabetes and hypertension and their views on the causation of these conditions. In most cases we also asked informants to recall what they had eaten in the previous 24h, and with whom they had eaten. Some informants responded to an invitation to ask their own questions by engaging in a discussion over the causation of diabetes and hypertension and asked for clarification of some of the health education messages they had received.

## Results and discussion

We have grouped the results of the interviews into a number of themes. Firstly we reflect on the insights offered on changes in food production systems, including the dominant crops grown, crops that are no longer grown and ‘new’ crops. Secondly, we present the findings on perceptions of dietary content and quality, food consumption patterns and questions of food security and insecurity. Lastly, we present some of the views expressed on the nature of the current food system in relation to the ‘new’ diseases such as Type 2 diabetes and hypertension.

### Food production and processing

Our interviewees were born and brought up in different parts of the country, under a variety of food production systems. The majority of informants interviewed in the urban Area 25 of Lilongwe had spent their childhoods in parts of rural Malawi where maize was and remains the dominant staple crop ^2^. By contrast, in rural Karonga district, we were interviewing in an area where cassava-growing has dominated. As might be expected, urban interviewees had generally experienced more change in food over their lifetimes, but it is important to note that change is not confined to that represented by a permanent move from rural to urban residence. Historically, many rural Malawians (especially those from the northern parts of the country) have migrated to work, either within the country or for work in Zimbabwe, South Africa or (to a lesser extent) Tanzania. Some of our interviewees clearly reflected this experience, commenting on the different kinds of foodstuffs they had consumed when working as wage labourers or as partners of wage labourers. Most older people, even when they have lived for decades in urban Lilongwe, are well versed in agricultural matters, particularly in those connected with food production, and when possible many maintained small plots of land at the margins of the city. For poorer households in particular, these plots of land were vital components of their present food supply.

Many of our informants recalled that when they were growing up their parents aimed at, and sometimes achieved, self-sufficiency in food production – though most also noted that there were marked seasonal variations in food supply. They reflected that this self-sufficiency had been based on the relative abundance of land – something that had changed radically with population growth since their childhoods. They typically contrasted this with the present-day situation in which “food depends on money”.

Most interviewees in urban Lilongwe recalled that, growing up in rural parts of the country, their parents had grown maize as a staple crop, along with beans, groundnuts and a variety of vegetables. The maize grown came from local varieties. Depending on origins, their families had supplemented maize with other cereal or starch crops, including cassava and sweet potatoes. Sometimes they had grown millet or sorghum ^3^. The latter two crops were mostly used for processing into beer. Many commented that the local maize varieties had been grown without fertiliser – something that had now become impossible due to the depletion of the soils – and could be more easily stored without the use of pesticide ( McCann, 2005; Smale, 1995). They talked about the new varieties of hybrid maize now being grown in the country, their reliance on chemical fertilisers and their susceptibility to insect infestation in storage. Some emphasised the importance of beans and other legumes (often intercropped with maize) in their childhood diets and spoke of the widespread cultivation and use of groundnuts, bambara nuts ( *mzama*) and pigeon peas ( *nandolo*) ( Katungi *et al.*, 2017). Many spoke of the use their families had made of a variety of wild foods, most of which were now unavailable due to population pressure and deforestation. These included wild loquats, figs, “ *matowa*”, a variety of wild roots vegetables, okra and wild mushrooms, as well as flying ants and caterpillars ^4^. 

In rural Karonga district we were interviewing close to the lake shore in an area where the production system has long combined fishing with cassava and rice production. Cassava remains the main staple in this area, along with supplementary crops of maize and rice, but fish stocks have declined significantly, and this was reflected in the interviews. ( Chipeta & Bokosi, 2013) All informants recalled their families growing ‘old’ varieties of cassava ( *mbundumali, thepula, mpapa* and *chitembere)* which did not require fertiliser. Many remembered that land had been abundant in their youth and that it had sometimes been possible to grow and store (in the ground) enough cassava to feed a family for two years. In the Karonga district interviews, informants also talked about growing a range of rice varieties, as well as the use of millet and sorghum (primarily for beer), older ‘red’ varieties of maize and bananas. Many dwelt on the importance of fish and (to a lesser extent) livestock in their childhood diets. One man, whose father had been a very active agriculturalist, talked at length about the importance of milk in his family’s subsistence, along with the fish that his father used to trap on the Songwe river (some distance to the north of our research area), and the maize grown along the river banks. In this family as in some others, use had been made of different plots of land with different qualities, including ‘upland’ land away from the lake for growing maize, and wet river side land for rice production. As was the case with the Lilongwe interviewees, most had strong recollections of the use made in the past of a variety of wild foodstuffs, as well as the past importance of legumes and groundnuts, the latter being still grown, but in less significant quantities.

Interviews with older people tend by their nature to draw attention to change, with reflections emerging on differences between past and present. In this case, many interviewees compared the abundance of land in the past with its relative shortage in the present, and consequent changes to food production systems and diet. Though the increased consumption of sugar did not form a major part of our discussions, many informants recalled that in the past sugar had been less widely consumed and was a feature of urban rather than rural diets. Some also reflected on increased salt consumption and recalled how in the past salt had sometimes been produced locally from potash-yielding plants. Salt had also been the subject of a set of powerful health-related beliefs, particularly in respect of women and children. Many informants were aware that increased salt consumption was linked to hypertension ^5^. A number of women informants dwelt at length on the subject of the present day use of purchased cooking oil, which they generally regarded as excessive. Some described how in the past groundnut flour has been more widely used in cooking, but that this had been replaced by the use of purchased oil. Women recalled how they had formerly made their own oils by hand, from groundnuts or fish, and used this for cooking. Informants in Karonga district remembered that the first commercial cooking oil to reach them had come via the trade with Tanzania.

But if change was highlighted by some, it is important to recognise that continuity was also a central part of these narratives. Most people felt that the essential element of the diet (the staple crop) had remained fairly constant. In the case of most interviewees in urban Lilongwe this meant maize; in the case of Karonga, cassava. Very few older people we interviewed felt that their diets had radically changed. However, they also stressed that within this apparent continuity there had been significant shifts. Firstly, they emphasised that though the staple crops had remained largely the same, the varieties of these crops grown had changed. In both maize and cassava-growing areas, new varieties introduced by government agents over time had largely (but not entirely) taken over from ‘local’ ones, and these new varieties were grown with fertiliser ^6^. Secondly, most individuals mentioned the decline of minor cereal crops (sorghum, millet), traditional vegetables (some cultivated, some wild) and the relative decline of legume production, including groundnuts. Several informants remarked that in the past they had grown a greater variety of foods, though they also remarked that now they knew about the “five food groups” (a mainstay of nutritional education in Malawi) whereas previously they had not. Thirdly, there was significant discussion on the question of food processing, particularly in relation to maize. Even where cassava varieties grown have changed, women described continuity in the processing of the plant for consumption. Cassava processing is notably labour intensive and though some attempts have been made to introduce mechanical milling of cassava in Malawi, none of our informants had experience of this. The processing of cassava in the past, as described by female informants, involved peeling the roots, soaking (in traditional clay pots), sun-drying and pounding. Most said that this process had not changed. By contrast, interviewees brought up in maize-producing areas reflected that the maize processing had changed radically with the spread (since around the 1940s) of maize mills. “In the old days”, said one woman, “we used to pound maize in a mortar and then soak it for a week so that it becomes soft and easy to pound. Nowadays we just take it to the mill”. Some informants emphasised that the more refined the maize flour, the more it was appreciated, so that in the past women might repeat this process of soaking and drying and pounding over three days to produce a fine flour. There was much discussion of the relative qualities of hand processed and milled flour, the latter being generally much less fine and more akin to ‘ *ngaiwa*’ , a rougher product incorporating bran. Though our interviewees in general preferred the more refined maize meal of the past, as a result of nutritional education many were also aware that ‘ *ngaiwa*’ was recommended as healthier, especially for those with type 2 diabetes or ‘sugar’ as it is called ^7^.

### Ways of eating, food security and hunger

Questions of food preparation led naturally into discussions of the organisation of eating and experiences of food security and hunger. Many informants, even those who held to possibly romanticised memories of the ease of food production in the past, stressed that this did not mean they have never experienced hunger. Indeed, most gave the impression that seasonal hunger was a commonplace experience, clearly distinct from enduring hunger leading to starvation. On the latter point, a number of informants in both locations recalled the events of the 1949 famine, which they had either experienced first-hand, or had been told about. But this was marked in their memories as an exceptional event. More commonplace was seasonal hunger emerged as a theme in many interviews, experienced by some, but not all rural households. ^8^One man in Karonga district, who had described the abundance of food produced by his family when he was a child, added “I should not lie, in those days we used to have hunger”. Like many others, however, he added that vulnerability to hunger had been partially offset by “a spirit of unity”, meaning that better-off families like his own would frequently invite poorer people to work on their land for them, and pay them in either raw foodstuffs or cooked *nsima.* This casual work, or *ganyu*, is still an essential part of the rural food economy, especially during food shortages. Seasonal hunger was also offset by the availability of wild foodstuffs, as noted above. One woman recalled that her father had had five wives and a large amount of land. Unlike some of their neighbours who regularly experienced hunger, they had been food secure. Nevertheless, she was able to list a large number of wild foods (roots, leaves) used in the past as a resource for hungry times. Many remarked that hunger now was caused by lack of money and by a decline in the spirit of sharing. However, one woman’s comment was a reminder of the existence of food insecurity in the past, and of its reproduction. Born in 1949 into a family with relatively little land, she had experienced hunger as a child, and continued to do so : “Let me say in short, the way I used to starve in the past at home is the same way I starve nowadays.”

Eating patterns and arrangements formed another strong theme in the interviews, and one on which many informants spoke at some length. Firstly, many people remarked with some consternation and humour on the current expectation of three meals a day. The comment, “in the past, there was no such thing as breakfast, lunch and dinner”, was repeated several times. Eating twice, or even once a day, had not been an unusual experience, and did not necessarily imply “hunger” as it would now. One woman, for example, commented that in the past “food was enough and could last longer, while today we have plenty of food but it doesn’t last long”. She went on to say that “in those days when you wake up in the morning you just take a banana, until 2 pm. Sometimes we only had lunch and did not have another meal until the next day”. This was echoed by other informants, many of whom mentioned ‘snacking’ in the morning on leftovers from the previous day’s meal, or a small piece of boiled cassava. Some implied that the lack of “three meals a day” was in part due to the “heavy job” of pounding maize and producing *nsima* (particularly at peak times in the agricultural cycle). “We couldn’t pound maize every day”, said one woman, so “we were eating what was available that day – we were cooking *khobwe* (cow peas) and pounded maize husks, *nkhowe.”* Similarly a man in Lilongwe remembered that cooked meals were limited in the rainy season, when everyone was busy on the fields.

This pattern of eating “whatever we could find” is still a marked feature of some of the households in which we conducted interviews, but appears now to be more closely associated with monetary poverty and the consequent inability to acquire food (especially maize flour) in bulk. : “What I can say is people are worse fed now and with very limited food choices since what enables them to have these foods is money and they just cook food enough for that particular day.” ^9^

If the pattern of meals had changed for some, so too had the social organisation of eating. Though experiences were far from uniform, most older informants felt that there had been a shift from more communal forms of eating, sometimes involving more than one household, to more “private” or individualised ways. Of course, some of this shift might reflect the fact of ageing, the accompanying reduction of household size and (in the case of Lilongwe residents) urban ways of living, but beyond this was a larger change. Many informants described eating in the past as a practice that was more communal than in the present, but simultaneously more hierarchical. ( Mandala, 2005; Vaughan, 1987) As children, most informants had been brought up in extended or polygynous families. Meals were frequently organised collectively, with a number of primary households contributing food. In this way, as many of our interviewees remarked, it was possible to share food with needy households at the point of consumption ^10^. As one woman (aged 84) in Karonga put it:

“In those days the whole family was eating together… also in those days if you didn’t have enough food you would ask a neighbour, no problem…. I think eating in groups like relatives was much better because if one family has no food it can be supported by other families.”

The same woman remarked that good cooking was highly valued and that as eating was communal, people would get to know which women were the best cooks. Another woman in Karonga commented that now everyone ate “privately” but previously there had been food sharing and “we were able to care for each other”. Another said that in the past “we could share food with neighbours, we were loving each other. Now people don’t love each other – money is the root of all evil.” As this remark implies, food sharing is seen by older people as emblematic of a moral order that has been undermined by the cash economy. Some remembered a time when eating alone was almost unheard of, since it implied a degree of selfishness associated with witchcraft.

Interviewees were clear, however, that though communal eating allowed for sharing and redistribution, formally its organisation was anything but egalitarian. Men, women and children ate in separate groups. Though strict food taboos appear to have been rare, many older people remarked that adult men received the choicest pieces of meat and fish. Almost everyone remembered that children were often relegated to eating whatever the adults had left at a meal - a memory echoed in the historical documentary record ^11^. Perhaps this memory is particularly acute now that so much emphasis is paid to infant and child nutrition in Malawi.

Though most informants remembered that the experience of seasonal hunger was very common in the past, nevertheless, nearly three quarters of them felt that in general people are less well fed today than they had been. This view appeared to be related not so much to the quantity of food available as to its quality. Perhaps this is a universal theme amongst older people – the sense that food tasted better in the past – and should therefore not necessarily be taken literally. However in this set of interviews it was also closely connected to a powerful theme around chemical contamination. Many of our interviewees expressed the view that production processes (particularly the use of chemical fertiliser and pesticides) was changing the character of their foodstuffs in ways that were detrimental to health. Some went so far as to imply that they were being poisoned by chemicals. This was most powerfully expressed in discussions of the use of pesticide on vegetables being sold in our informants’ communities. Older people are well aware that strong pesticides are applied to, for example, green leaf vegetables, which are then sold immediately afterwards for consumption. They also made a less direct link between fertiliser use and unhealthy food. Some expressed the view that the ‘speeding up’ of growth of foodstuffs (staple crops, vegetables and also livestock such as chickens) was artificial and unhealthy. This in turn they linked to the rise of the ‘new diseases’ of hypertension, type 2 diabetes and other conditions such as asthma. One man put it succinctly like this:

“There are many problems, though I don’t have wider knowledge but I think that asthma and BP they are caused by fertiliser because fertiliser is made up of chemicals, and the fact that hybrid varieties of crops have been introduced, they want us to grow in a quick manner like hybrid crops! [laughs]”.

This does not imply that they were unaware of health education messages around diet and exercise in relation to NCDs – indeed many of them were attempting to follow that advice – but it is indicative of a vernacular knowledge that links health to contamination and exposure, itself a subject of increasing medical scientific research.

## Conclusion

A small scale study such as this clearly has severe limitations, but it does point towards possible topics for future research. Older Malawians see both change and continuity in their diets. At one level, the ‘typical’ Malawian meal of a staple porridge accompanied by relish remains a constant of the diets of older people in both urban and rural areas. The industrialised food that increasingly dominates diets in some parts of southern Africa did not appear to feature much in their lives or in their accounts. However, our interviewees were well aware that beneath the apparent continuity there was change. Comparing present day diets to those of their childhoods, many informants pointed to the decrease in diversity of food crops grown, largely due to population pressure and declining soil fertility. More than ever, they argued, the ability to feed oneself and one’s family adequately was dependent on access to cash. Our informants were realists and pragmatists. They could see improvements in the food system (more egalitarian distribution at mealtimes, less female labour taken up in manual processing) as well as disadvantages. Most were well aware of the increasing problem of diet-related illnesses in their communities, especially hypertension and type 2 diabetes, and many of them made an effort to follow the dietary advice given to them by health workers. However, a strong theme emerged from these interviews with older people which implies a suspicion of modern methods of production and food processing. This is the suspicion that the chemicals (fertiliser and pesticides) on which the food system depends are themselves harmful to health and a driver of the ‘new’ diseases. Also evident from this study is the fact that many older people have a valuable store of food knowledge, particularly in relation to wild plants and forgotten cultivars, which is likely to be lost very soon unless recorded and documented.

## Data availability

The original data is held at the Malawi Epidemiology and Intervention Research Unit, PO Box 148, Lilongwe, Malawi. Requests for access can be made to the Director of Malawi Epidemiology and Intervention Research Unit: mia.crampin@lshtm.ac.uk. Data will be released under the condition that the anonymity of the individuals involved in the study is preserved and that acknowledgement is made to the original research study.

## Footnotes

1 The Lancet Commission on NCDI Poverty is due to issue its report on Malawi soon: www.ncdipoverty.org

2 Maize is not a ‘new’ crop in Malawi though it is sometimes referred to as such. It has been cultivated in the region from at least the late eighteenth century, but its importance in relation to other staple crops (millet and sorghum in particular) varied according to environmental conditions. It was introduced from the New World via the east coast of Africa through the long-distance trade networks that crossed the lake Malawi region in the eighteenth and nineteenth centuries. Maize undoubtedly became more dominant in the course of the twentieth century. The colonial government encouraged its production as a cash crop and introduced a centralised maize marketing system, though colonial administrators also encouraged cassava as a ‘famine’ crop. In Malawi maize is now often referred to as *the* staple food. An oft-quoted phrase is ‘ *chimango ndi moyo*’ meaning ‘maize is life’, and this sense of the importance of maize has probably increased with the introduction of hybrid maize and the targeted subsidies that now accompany production. The size of the annual maize crop is a direct indicator of the country’s food security. Undoubtedly maize has been a very successful crop in this region for at least two hundred years, but it is important to note that in living memory it was often grown as part of a ‘bundle’ of staple crops. Many of our informants refer to this. McCann, 2005; Mzama, 2011; Smale, 1995; Vaughan, 1987)

3 On the recent history of sorghum On the recent history of sorghum and finger millet in northern Malawi see Bezner Kerr, 2014

4 Wild foods of various kinds are still widely consumed in Malawi, but our informants pointed out that their availability had been greatly reduced by the increase in population density. Wild foods have been critical to survival in periods of famine: Vaughan, 1987; Williamson, 1974

5 Sodium-based salt was a relatively rare and valued commodity in the nineteenth century, traded along long distance networks from the coast. The production of potash plant-based salt was documented by colonial researchers – see especially, Williamson, 1956. On salt taboos and child health: Morris, 1996

6 On the adoption of new varieties of maize see Smale, 1995; Smale *et al*., 1995

7 On glycemic responses to different kinds of maize flour consumed in Malawi see Mlotha *et al*., 2016. The relative decline of soaking in the processing of maize meal may have nutritional consequences: Holz & Gibson, 2007

8 Seasonal hunger was clearly documented by the Nyasaland Nutrition Survey in the late 1930s and early 1940s, and remains a feature of rural life in Malawi today despite the extensive commercial market in staple foods. On the history of hunger in Malawi see Mandala, 2005

9 See also Ene-Obong & Odenigbo, 2015

10 This method of food redistribution and support for poorer households was noted by colonial researchers. See also Vaughan, 1983

11 Researchers on the Nyasaland Nutrition Survey found it particularly hard to quantify the consumption of young boys since they were so often absence from family meals and were to some extent expected to fend for themselves by hunting, gathering wild foodstuffs and ‘begging’. The anthropologist, Audrey Richards, recorded a similar phenomenon in her study of the Bemba diet in Northern Rhodesia (Zambia) : Richards, 1995
