# Supplementary material for: Dietary change, noncommunicable disease and local knowledge: results of a small-scale study of the views of older Malawians
Source: Wellcome Open Res. 2018 Dec 11;3:158. [Version 1] doi: 10.12688/wellcomeopenres.14887.1 (PMC6338127; doi:10.12688/wellcomeopenres.14887.1)
Supplement: Peer review history

# Peer review: Referee response for version 1

**DOI:** 10.21956/wellcomeopenres.16228.r34431

**Published:** 2019-01-15

**Authors:** Chisomo Kalinga

**Affiliations:** 1 Centre of African Studies, University of Edinburgh, Edinburgh, UK

This is an excellent article, which clearly establishes parameters and influencing factors that guided the selection of limited number of participants. 

 The article establishes a compelling presentation on perceptions of food and dietary change in order to investigate how local understandings of food are unique to each elderly participant and his/her personal and historical conceptualization of food and its correlation to health. Yet it also articulates how they also connect to broader issues facing communities and their understanding of food, food security, NCDs and perceptions of health across Malawi. The authors' assertion that this study should be a starting point to further in-depth research is appreciated. 

 In that vein, it would be interesting to see the authors expand upon this in the future to include 1) specific and nuanced local histories of food and indigenous perceptions of their relationship to health and 2) incorporates indigenous knowledge transmission practices regarding nutrition and their survival alongside modern agricultural procedures. 

 This investigation advocates the importance of comparative studies amongst regions and the potential for comparative sets of data to reveal trends in food production, consumption and household compositions in who produces, procures and prepares food. This model of study would provide rich data to contextualist local knowledges of NCDs and perceptions of dietary change and variations of thought across Malawi. In particular, it would be interesting to understand *if* and *how* the roles of women in food production and perceptions of diet and health, particularly in the Karonga rice-growing region, contributes to observed differences in the production, distribution, and vending of crops compared to other regions. 

 In conclusion, this is an important piece of scholarship that should inspire more studies into the intersection between technological advancement (includes but not limited to GMOs, fertilization and pesticides) and local histories of food production and their collective influence on perceptions of health and NCDs.

I have read this submission. I believe that I have an appropriate level of expertise to confirm that it is of an acceptable scientific standard.

# Peer review: Referee response for version 1

**DOI:** 10.21956/wellcomeopenres.16228.r34433

**Published:** 2019-01-14

**Authors:** Wapulumuka Mulwafu

**Affiliations:** 1 Department of History, Chancellor College, University of Malawi, Zomba, Malawi

**Is the work clearly and accurately presented and does it cite the current literature?**

 This paper is well written and focuses on a very topical issue concerning the growing burden of non-communicable diseases in Malawi and the sub-Saharan region. From the perspective of both social scientists and health professionals, the study adds a new dimension to the knowledge and understanding of the relationship between dieting and NCDs. The paper has engaged adequately with current literature on the subject matter and demonstrates how this particular approach of studying dietary changes over time will contribute to new knowledge on occurrence of NCDs.

**Is the study design appropriate and is the work technically sound?**

 Being an exploratory study, the design is appropriate and well conceptualised. The choice of both urban and rural areas for data collection presents a perfect opportunity for comparing the relationship between dietary changes and the occurrence of NCDs. However, the Malawi Epidemiology and Intervention Research Unit, in whose research areas this study was conducted, may have had good reasons for choosing Karonga and Lilongwe, but it is not very clear why this study decided to work in the same research sites. Perhaps a short explanation showing this connection would be necessary.

**Are sufficient details of methods and analysis provided to allow replication by others?**

 The study methods are clearly explained in the paper and provide a good basis for replication by other researchers. There is also a well thought out summary of key issues covered in the research guide and this gives readers a chance to appreciate the process which researchers used to collect and analyse the data.

**If applicable, is the statistical analysis and its interpretation appropriate?**

 This study is purely qualitative and did not use any statistical analysis. But the data generated have been thematically analysed and I find the interpretations to be contextually appropriate.

**Are all the source data underlying the results available to ensure full reproducibility?**

 The authors have indicated that all the data relating to this research will be kept at the Malawi Epidemiology and Intervention Research Unit in Lilongwe. The informants have been anonymised but the data can be accessed by any interested person to ensure reproducibility of the results.

**Are the conclusions drawn adequately supported by the results?**

 The authors are very clear about the limits of their study and yet the findings coming out of this research are illuminating. The conclusions are well supported by evidence from the results. This makes it all the more necessary to encourage the team to conduct a full scale research whose findings could have important scholarly and policy implications.

**Minor typographical errors:**

 Type 2 diabetes: need to write this consistently throughout the paper. In some cases the word “Type” is capitalised and in other cases it is not.

 Page 4, column 2, last but one paragraph, line 3 from bottom: check the spelling of “matowo”.

 Page 4, footnote 3: “on the recent history of sorghum....” phrase is repeated.

I have read this submission. I believe that I have an appropriate level of expertise to confirm that it is of an acceptable scientific standard.

# Peer review: Referee response for version 1

**DOI:** 10.21956/wellcomeopenres.16228.r34434

**Published:** 2019-01-07

**Authors:** Rachel Bezner Kerr

**Affiliations:** 1 Department of Development Sociology, Cornell University, Ithaca, NY, USA

This paper provides exploratory analysis of an under-researched area in Malawi, namely elderly people's view of dietary changes. While it is a small sample, it provides a preliminary assessment of dietary changes, and perceptions of these changes, and points to potential future research. The results are relevant for understanding some of the changes observed over several generations. The one limitation is that more details on specific findings would help shed light into the conclusions, including more detailed quotes. One example is the brief reference to gathered wild foods, and the loss of knowledge which respondents noted - more specific details of these findings would strengthen the conclusions. This is a minor issue, however, and in general the article is well done.

I have read this submission. I believe that I have an appropriate level of expertise to confirm that it is of an acceptable scientific standard.
